# Do Cells Contribute to Tendon and Ligament Biomechanics?

**DOI:** 10.1371/journal.pone.0105037

**Published:** 2014-08-15

**Authors:** Niels Hammer, Daniel Huster, Sebastian Fritsch, Carsten Hädrich, Holger Koch, Peter Schmidt, Freddy Sichting, Martin Franz-Xaver Wagner, Andreas Boldt

**Affiliations:** 1 Institute of Anatomy, University of Leipzig, Faculty of Medicine, Leipzig, Germany; 2 Institute of Medical Physics and Biophysics, University of Leipzig, Faculty of Medicine, Leipzig, Germany; 3 Institute of Materials Science and Engineering, Chemnitz University of Technology, Chemnitz, Germany; 4 Institute of Forensic Medicine, University of Leipzig, Faculty of Medicine, Leipzig, Germany; 5 Translational Centre for Regenerative Medicine, University of Leipzig, Faculty of Medicine, Leipzig, Germany; 6 Institute of Sport Science, Department Human Locomotion, Chemnitz University of Technology, Chemnitz, Germany; 7 Institute of Clinical Immunology, University of Leipzig, Faculty of Medicine, Leipzig, Germany; Bascom Palmer Eye Institute, University of Miami School of Medicine, United States of America

## Abstract

**Introduction:**

Acellular scaffolds are increasingly used for the surgical repair of tendon injury and ligament tears. Despite this increased use, very little data exist directly comparing acellular scaffolds and their native counterparts. Such a comparison would help establish the effectiveness of the acellularization procedure of human tissues. Furthermore, such a comparison would help estimate the influence of cells in ligament and tendon stability and give insight into the effects of acellularization on collagen.

**Material and Methods:**

Eighteen human iliotibial tract samples were obtained from nine body donors. Nine samples were acellularized with sodium dodecyl sulphate (SDS), while nine counterparts from the same donors remained in the native condition. The ends of all samples were plastinated to minimize material slippage. Their water content was adjusted to 69%, using the osmotic stress technique to exclude water content-related alterations of the mechanical properties. Uniaxial tensile testing was performed to obtain the elastic modulus, ultimate stress and maximum strain. The effectiveness of the acellularization procedure was histologically verified by means of a DNA assay.

**Results:**

The histology samples showed a complete removal of the cells, an extensive, yet incomplete removal of the DNA content and alterations to the extracellular collagen. Tensile properties of the tract samples such as elastic modulus and ultimate stress were unaffected by acellularization with the exception of maximum strain.

**Discussion:**

The data indicate that cells influence the mechanical properties of ligaments and tendons in vitro to a negligible extent. Moreover, acellularization with SDS alters material properties to a minor extent, indicating that this method provides a biomechanical match in ligament and tendon reconstruction. However, the given protocol insufficiently removes DNA. This may increase the potential for transplant rejection when acellular tract scaffolds are used in soft tissue repair. Further research will help optimize the SDS-protocol for clinical application.

## Introduction

Acellular scaffolds are increasingly applied in soft tissue reconstruction or in the surgical treatment of musculoskeletal system injury [1]–[5]. The rationale behind acellularization is to provide hetero- or xenogenic tissues that cause reduced immune response [3], [6]. An additional aim is to slow down physiological degradation and to enhance ingrowth of the body's own cells. For this purpose, the scaffolds are chemically cross-linked [3], [7], [8] or seeded with growth factors [9]. A diversity of acellular tissues is used for this purpose, such as dermis [5], [10]–[12], pericardium [7], [13], ligaments and tendons [6], [14] or small intestine submucosa [3], [5], [15]. These tissues mostly originate from human, bovine, caprine, equine or porcine donors [15].

In the current literature there is a variety of biomechanical and histological descriptions of acellular scaffolds using *in-vivo* [6], [7], [10] and *in-vitro* models [1], [5], [16], [17]. However, many of these studies are based on small sample sizes or are associated with large measurement variations [1], [7]. Most of these studies also lack standardized testing conditions [6], [10], [11] or sampling conditions [1], [4], [5]. As a consequence, the results from mechanical testing are not readily comparable. Such a comparison, however, would help determine the suitability of the various scaffolds for the differing anatomical target sites from an anatomical point of view. No previous studies have aimed to quantify the effect of the acellularization procedure by directly comparing tissues obtained from the same human donors, at the same anatomical site, before and after acellularization. Doing so would help to gain insight into the influence of cells on connective tissue biomechanics, which is the aim of our study. Furthermore, such comparison would help to estimate the effects of the chemicals applied for acellularization on the mechanical properties of collagen tissues.

We aimed to establish a protocol for a direct comparison of the tensile properties of native samples and their acellular scaffold counterparts, obtained from the same donor and the same anatomical site. The protocol should specifically circumvent the following four sources of bias, as identified in the literature, which could potentially impact on the results of mechanical testing:

1. Differences in the tensile properties of related tissues originating from different species, different donors or different anatomical sites - by comparing the mechanical properties of samples obtained from the same donors and the same anatomical sites.
2. Lack of standardized testing conditions - by obtaining uniaxial tensile properties of tissues that are mainly loaded uniaxially.
3. Altered water content related to storage or acellularization [18], and its impact on the tensile properties - by using the osmotic stress technique to adjust the samples' water content.
4. Material slippage - by minimizing slippage using the partial plastination technique [19].

To check the validity of the protocol and gain insight into the influence of cells on the mechanical properties of collagen tissues, the following hypothesis was addressed:

Cell loss alters tensile properties of human ligaments or tendons such as the human iliotibial tract *in vitro*.

## Material and Methods

### 1. Human iliotibial tract preparation

**Figure 1:**
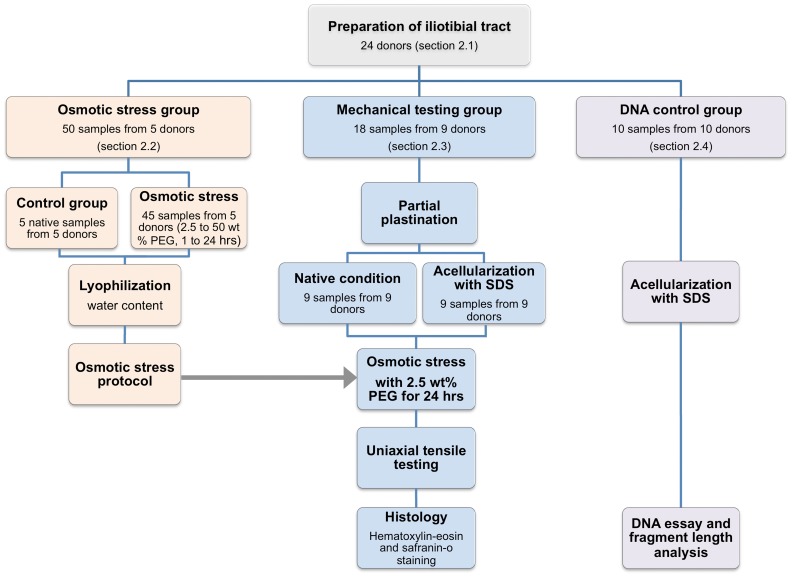
Summary of the study protocol and the amount of specimens that were used for the respective experiments.

Twenty-four human iliotibial tract specimens were obtained from twenty-four body donors (6 females, 18 males, age 33.7±16.2 years; Table S1 in File S1) during autopsy at the Institute of Forensic Medicine, University of Leipzig, Germany. The donors had no history of connective tissue disease and the tissues were removed in a fresh and chemically unfixed condition. The university's ethics committee “Ethik-Kommission an der Medizinischen Fakultät der Universität Leipzig” approved the study (protocol number 156-10-1207-2010). The ethics committee waived the need of written consent from the donors. If possible, written consent was obtained from the donors' relatives. A longitudinal incision measuring approximately 15 cm in length was made, starting at the greater trochanter, directed to the lateral epicondylus of the respective side. Following the incision, the iliotibial tract was visualized and removed in the region of most parallel fibers. The tracts were immediately freed from surrounding tissues under constant moistening with isotonic sodium chloride solution, precooled at 3°C and shock frozen at −85°C for storage. Tract specimens of five donors were used for determining the water content in the fresh condition and for establishing a protocol that allows adjusting the water content of tract specimens after acellularization to their initial value (*osmotic stress group*). Nine tract specimens were tested mechanically in the native and the acellular condition before they were used for histology (*mechanical testing group*). The remaining ten tract specimens were used to determine the DNA content after acellularization (*DNA control group*). Figure 1 summarizes the study protocol and the amount of specimens that were used for the respective experiments.

### 2. Osmotic stress group: establishing an osmotic stress protocol to adjust samples' water content to their initial value

**Figure 2:**
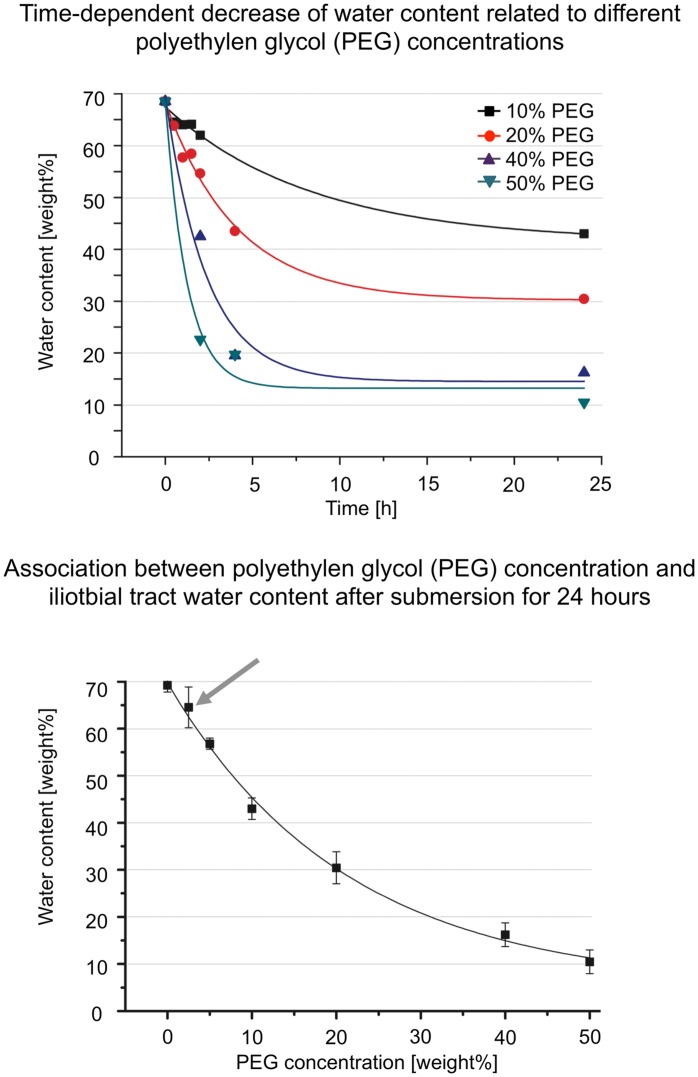
Time-dependent decrease of the water content as a function of the polyethylene glycol (PEG) concentrations (2a, top) and correlation between polyethylene glycol (PEG) concentration and iliotbial tract water content after submersion for 24 h (2b, bottom) are shown. Applying the osmotic stress technique to iliotibial tract specimens caused a PEG- dependent decrease of their water content. PEG concentrations of 2.5 wt% (grey arrow) were most suitable for osmotically adjusting the water content of iliotibial tract samples and their corresponding acellular scaffolds.

In a first step, a protocol was established for adjusting the water content in the tract samples to their initial (native) value by means of the osmotic stress technique [20], [21]. For this purpose, polyethylene glycol (PEG; Rotipuran, Carl Roth GmbH + Co. KG, Karlsruhe, Germany; molecular weight 20,000 Da) was prepared in concentrations of 2.5 wt% (0.75 MPa) to 50 wt% (9.6 MPa; [22]) in an isotonic sodium-chloride solution, buffered with 20 mM tris (hydroxymethyl aminomethane; pH = 7). Five tract specimens from five body donors (Figure 1, Table S1 in File S1) were sectioned into ten samples each. Forty-five of the samples (9 samples/donor) were packed in 42-mm dialysis membranes (Carl Roth GmbH + Co. KG, Karlsruhe, Germany; molecular weight cut off  = 6000–8000 Da), sealed at both ends and submersed in the different PEG solutions for 1, 2, 4 and 24 h at 4°C under continuous stirring of the fluid (Figure 2). After the given times, the samples were weighed, lyophilized for 48 h and again weighed to measure their water content. The remaining five samples (1 sample/donor) served as controls to determine the initial water content of the iliotibial tract in the fresh condition [21].

### 3. Mechanical testing group

#### 3.1. Partial plastination of tract samples to optimize clamping in material testing

Eighteen samples (nine sample pairs) were harvested from nine tract specimens of nine donors by means of longitudinal sections made in the direction of the fibers (Figure 1; Table S1 in File S1). Sample thickness was left unaltered.

The ends of the 18 samples were freeze-substituted in acetone and primed with polyurethane resin, prepared in a ratio of 1/1/3 with RENCAST FC52 Isocyanate/FC52 Polyol/Ceramic Powder (RenShape solutions, Huntsman International LLC, Salt Lake City, USA; YM  = 2100 N/mm^2^). The resin was reinforced with Pertinax plates (PF CP 201, Dr. Müller GmbH, Ahlhorn, Germany) to improve the stability of the plastinated parts of the tract specimens that were to be clamped in the material testing machine. Gelatin and a template were used to protect the central unfixed part of the specimen from the acetone and from the resin. After polymerization of the resin, the specimens were rinsed in 40°C water to remove the gelatin and then immediately stored in isotonic sodium-chloride solution. The central parts of the samples remained in the unfixed fresh condition at all times, sharply separated from the resin of the plastinated parts. The samples measured 69.7±2.3 millimeters in length and ten or more millimeters in width. For more details on the partial plastination technique, please refer to [19]. One sample of each corresponding pair was again precooled and shock frozen, the other samples were subjected to acellularization.

#### 3.2. Acellularization procedure with sodium dodecyl sulphate (SDS)

The procedure of acellularization was accomplished as described previously [8]. Nine of the 18 partially plastinated samples to be used for material testing were submersed in an SDS solution (1% by volume; Roth, Karlsruhe, Germany) for seven days to remove all cellular components. The acellular scaffolds were then rinsed in distilled water for seven days. Both the SDS solution and the distilled water were exchanged once daily. After completing the rinsing, the nine partially plastinated scaffolds were precooled and shock frozen. Furthermore, ten acellular scaffold samples were forwarded to the DNA assay for comparison to their native counterparts.

#### 3.3. Adjusting tract samples' water content with the osmotic stress technique

**Figure 3:**
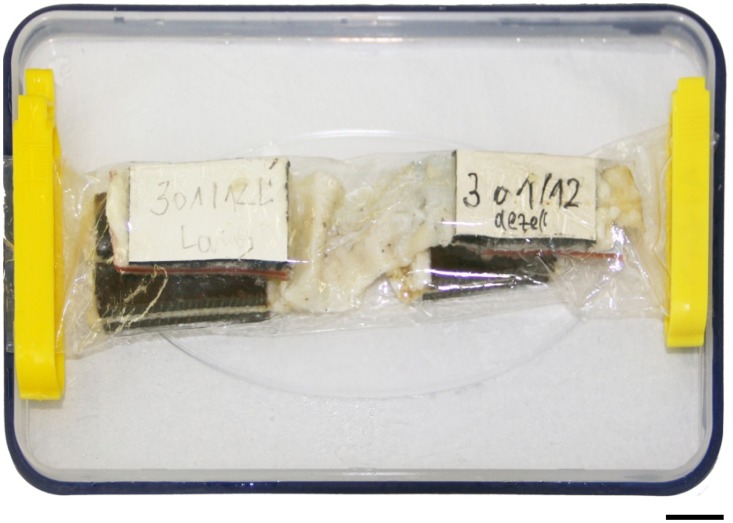
The osmotic stress technique was applied to partially plastinated iliotibial tract samples. Each sample pair consisting of one native tract and its corresponding acellular counterpart were submersed for 24 h in a 2.5 wt % polyethylene glycol solution before uniaxial tensile testing. Scale bar  = 10 mm

On the basis of the protocol established in the osmotic stress group, the osmotic stress technique was applied to adjust the water content to 69 wt% in all partially plastinated samples of the mechanical testing group (Figures 1 and 2). The corresponding sample pairs consisting of one native sample and one acellular scaffold were each packed and sealed in one dialysis membrane (Figure 3) and equilibrated in 2.5 wt% PEG solution at an osmotic pressure of 0.75 MPa for 24 h before material testing started.

#### 3.4. Mechanical testing

**Figure 4:**
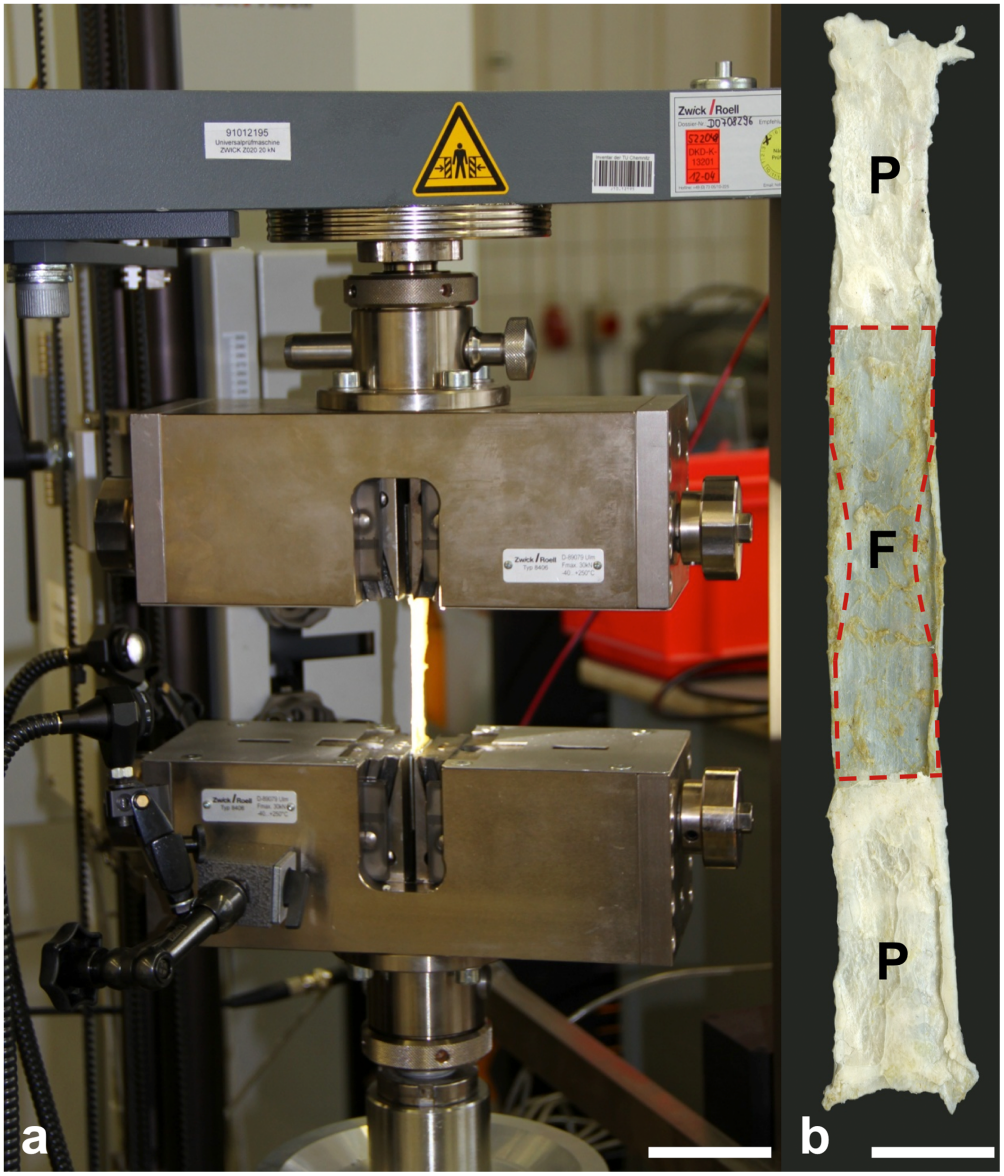
Experimental setup for uniaxial stress-strain testing of an acellular iliotibial tract sample (4a) and partially plastinated tact sample (4b) with a sketch of the template used to taper the specimens (red figure with broken line). F =  fresh (unfixed) and P =  unfixed part of the tract sample; scale bar  = 50 mm (4a), 17 mm (4b)

Elongation of the 18 partially plastinated samples was measured with a 5 kN load cell during uniaxial tensile deformation with a Z20.0 uniaxial testing machine (Zwick GmbH & Co. KG, Ulm, Germany; Figure 4a) at a temperature of 21°C. Both the acellular samples and their native counterparts, serving as the controls, were tested in the same manner. The samples were removed from the PEG solution and the dialysis membrane immediately before material testing, subsequently mounted and then tapered in a dogbone-like shape using a template to create a minimum cross-sectional area. The smallest distance of the taper measured 10 mm in width. Initial specimen thickness was recorded with an electronic micrometer (Universal Micrometer 0–25 mm, Werkzeug-Eylert GmbH, Chemnitz, Germany; resolution 1 µm, accuracy ±2 µm) under a preload of 10 N. Specimens' tapered and preloaded width was recorded with an Aramis system (GOM - Gesellschaft für Optische Messtechnik mbH, Braunschweig, Germany). Figure 4b shows a partially plastinated specimen in its original dimensions and the resulting dimensions after tapering. The specimens' cross-sections were calculated on the basis of the width and thickness, presuming a rectangular shape. Prior to a final cycle until material failure, each sample was preconditioned for 20 cycles at a load range of 10–100 N and at a crosshead displacement speed of 20 mm/min. Elongation of each sample was measured by the displacement of the crosshead co-registered by the digital image correlation method (Aramis, GOM - Gesellschaft für Optische Messtechnik mbH, Braunschweig, Germany; capturing rate 4 fps). Deviating from our previous experiments [19], [23], the samples were not moistened during the testing to keep the water content on the same level in all samples. The elastic modulus was determined by means of regression analysis in the most linear region of the stress-strain curve. Ultimate stress (stress at failure) was defined as the maximum stress before failure. Maximum strain was the engineering strain calculated from the ratio of elongation immediately before material failure and the initial length of the sample.

#### 3.5. Histology

For histological examination, the small tissue pieces were removed from the nine partially plastinated sample pairs immediately before the material testing, dehydrated in ascending ethanol series and then embedded with paraffin. Serial sections of 10 µm were stained with hematoxylin-eosin (HE) and with safranin-o staining [24]. Three investigators without prior knowledge to the allocation of the sections scanned the tissues for the presence of cell nuclei and analyzed the appearance of the extracellular collagen. To this end, at least three slices with ten randomly selected fields were investigated under 400-fold magnification from each specimen [25].

### 4. DNA control group: quantification of DNA removal by means of a DNA assay and qualitative fragment analysis with gel electrophoresis

Thirty samples were harvested from ten iliotibial tracts of ten donors to determine the effectiveness of the acellularization with SDS (Figure 1; Table S1 in File S1). Twenty of the samples (2 samples/donor) were acellularized with SDS as described above. After acellularization, ten of the samples (1 sample/donor) were additionally digested enzymatically for 12 h at 37°C with DNAse (200 µg/mL; Sigma, Deisenhofen, Germany) in isotonic PBS, containing 50 mM MgCl_2_. Ten samples were left in the fresh condition (1 sample/donor) and served as controls.

A standard DNA assay (DNeasy Blood & Tissue Kit, Qiagen, Hilden, Germany) was used to determine DNA content of the tract specimens before acellularization, after acellularization and after acellularization and DNA digestion, as proposed by Pridgen et al. [16] and Schulze-Tanzil et al. [9]. For this purpose, small pieces of the samples (20–30 mg) were incubated in a shaking water bath (56°C) for 12 h, containing lysis buffer and proteinase K. The DNA was purified and measured spectrophotometrically with a Nanodrop spectrophotometer (Peqlab, Erlangen, Germany). On the basis of the DNA content recorded from each of the native, acellular or acellular and digested samples, the extent of DNA removal was calculated as a ratio acellular/native tract or acellular and digested/native tract, as proposed by Pridgen et al. [16] and Schulze-Tanzil et al. [9]. For qualitative fragment length analysis, 5–12 µg of total DNA was electrophoretically, separated in a 1.5% agarose gel (50 min, 120 V). After the run, the gel was documented by light exposure in the FastGene GelPic LED Bos (Nippon Genetics Europe, Dueren, Germany).

### 5. Data analyses

The anthropometric data from the body donors, the results from mechanical testing and from the DNA assay were analyzed by means of Microsoft Excel version 2010 (Microsoft Corporation, Redmond, WA, USA) and SPSS version 20 (Chicago, IL, USA). The Kolmogorov-Smirnov test was used to determine normal distribution of the data. The Student's t-test was used to compare the mechanical data (elastic modulus, ultimate stress, maximum strain). The Tamhane test was used to compare the anthropometric data to the specimen's gender and side statistically. *P* values of 5% or less were considered as statistically significant. Bland-Altman plots were used to compare the alteration of the mechanical data caused by acellularization to the interindividual differences by means of GNU R (R Development, Auckland, New Zealand; [26], [27]). Mean differences, upper and lower limits of agreement (LOA) were calculated.

## Results

Comparison of the anthropometric data from the body donors revealed that their age, body length and weight did not significantly vary among the specimens used for the osmotic stress experiments, the mechanical testing and the DNA assay.

### 1. 24-h submersion in 2.5 wt% PEG solution allows adjusting the water content of tract samples to the initial value

The mean water content of the native samples was 69±1%. Increasing submersion times in the PEG solutions at the given concentrations resulted in decreasing water content of the tract samples. Their water content asymptotically approximated a final value reached after 24 h or less in exponential functions, as shown in Figure 2a. The initial decrease in water content was larger at higher PEG concentrations, i.e. the water content was 62±4% and 54±8% after submersion for 2 h in 10 wt% and 20 wt% PEG, respectively. Half-life time of water decrease was 1.2, 2.4, 4.1 and 8.4 hours for PEG concentrations of 50 wt%, 40 wt%, 20 wt% and 10 wt%, respectively. After 24 hours of submersion, the final water content of the samples was 10±3%, 16±3%, 30±3%, 43±2%, 57±1% and 65±4% at PEG concentrations of 50 wt%, 40 wt%, 20 wt%, 10 wt%, 5 wt% and 2.5 wt%, respectively (Figure 2b).

Equilibration of the tract samples in 2.5 wt% PEG solution providing an osmotic pressure of 0.75 MPa resulted in the closest water content, as compared to the native samples. A standard exponential fit equation was used to calculate the shortest submersion time to reach a final water content of 65% in 2.5 wt% PEG after 10.6 hours. All partially plastinated sample pairs were therefore dialyzed in 2.5 wt% PEG concentration for 24 hours prior to material testing.

### 2. Tensile material properties are unaltered in acellular tract scaffolds with the exception of maximum strain

**Figure 5:**
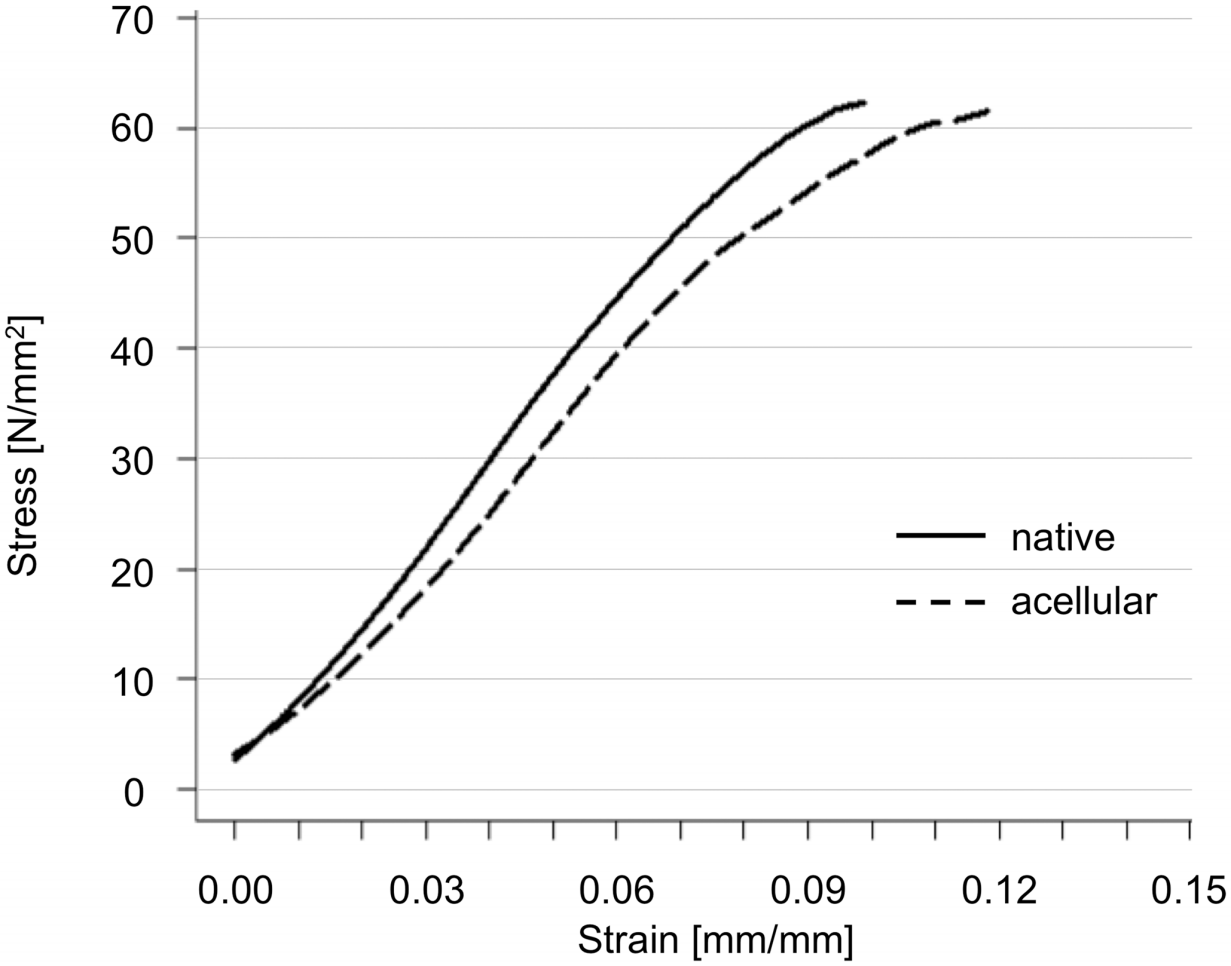
Stress-strain curves of native and acellular iliotibial tract samples obtained from the same donors and anatomical site.

**Figure 6:**
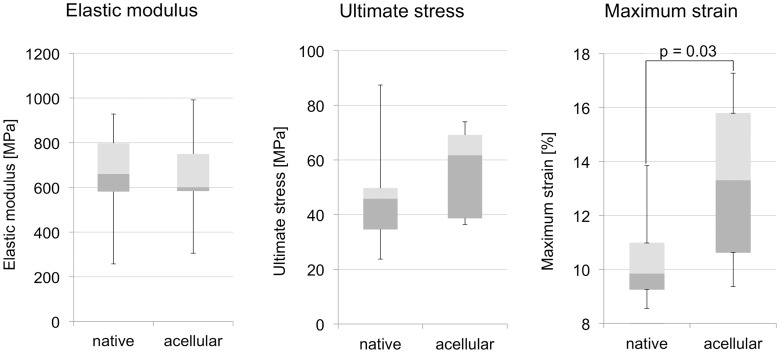
Box plots are depicted for elastic modulus (6a, left), ultimate stress (6b, center) and for maximum strain (6c, right). Material properties were unaffected by acellularization with the exception of maximum strain.

Stress-strain data of 18 tract samples were recorded. All specimens were tested until material failure. The failure site was always located centrally with one exception in the native and another in the acellular group, but without any signs of avulsion phenomena caused by the plastination resin. One representative stress-strain curve is depicted in Figure 5. The results of the data are summarized in Figure 6, in Table S2 in File S1 and in Figures S1 and S2.

The elastic moduli were 694±139 MPa and 652±199 MPa in the native and the acellular group, respectively (Figure 6a). The elastic moduli did not vary significantly between both groups (*p* = 0.41). Ultimate stress was 47±19 MPa and 52±14 MPa in the native and the acellular group, respectively (Figure 6b). Ultimate stress did not vary significantly between both groups (*p* = 0.26). Maximum strain was significantly larger in the acellular group (10±2%) than in the native one (13±3%; *p* = 0.005; Figure 6c).

### 3. SDS acellularization causes a near-total removal of cells but only a subtotal removal of DNA in the tract scaffolds

**Figure 7:**
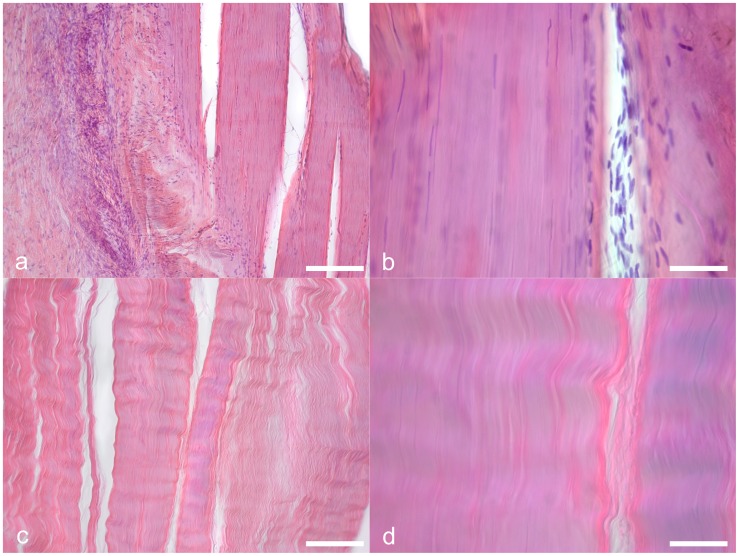
Hematoxylin-eosin stained histology samples were obtained from the native iliotibial tract and from acellular scaffolds. In the native samples (7a,b), nuclei are stained in an intensive blue color. In the acellular scaffolds, the nuclei vanished and the collagen appears to be washed out (7c,d). Scale bar 25 µm (7a,c) and 12 µm (7b,d).

**Figure 8:**
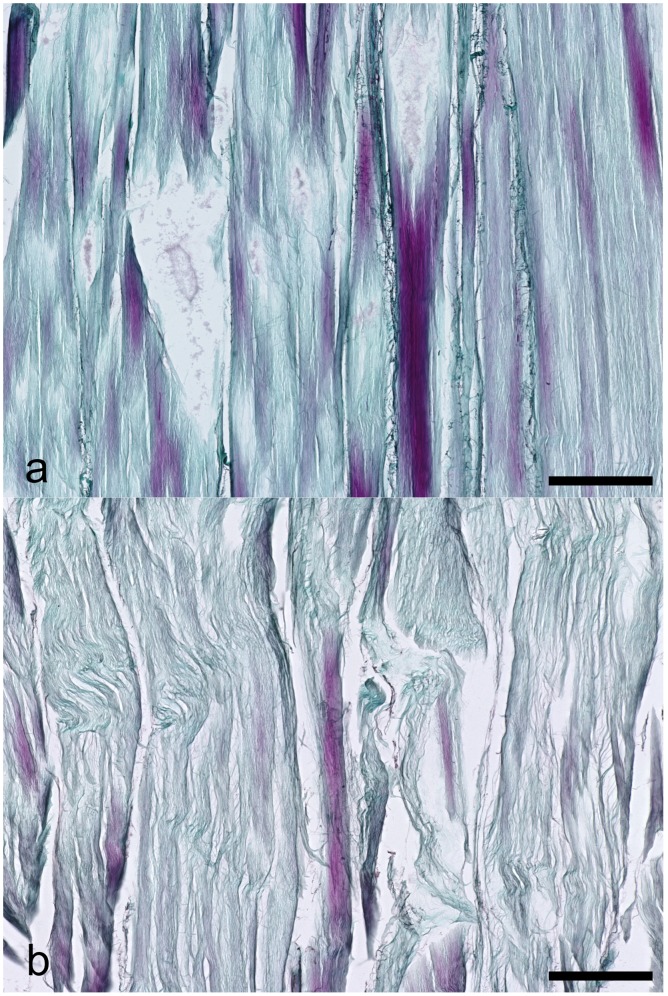
Safranin-o staining of native (8a) and acellular (8b) iliotibial tract samples. Scale bar  = 1000 µm.

All HE-stained samples obtained from all partially plastinated tracts could either be allocated to the native or to the acellular group by means of histological investigation. Inter-rater reliability of the allocation to either group was 100%. In the native group, the nuclei of the fibrocytes and of the vascular endothelium were stained in an intensive blue color, as shown in Figure 7a and b. The collagen fiber bundles separated sharply from the surrounding tissues. In contrast, no nuclei were observed in any of the acellular samples (Figure 7c,d). Here, the collagen fiber bundles appeared to be washed out, leaving behind blurry structural borders to the adjacent tissues. The safranin-o stained samples showed that the proteoglycans of the tract samples were preserved to a variable extent in both the native and the acellular samples (Figure 8). No changes that were clearly attributable to the acellularization were observed.

**Figure 9:**
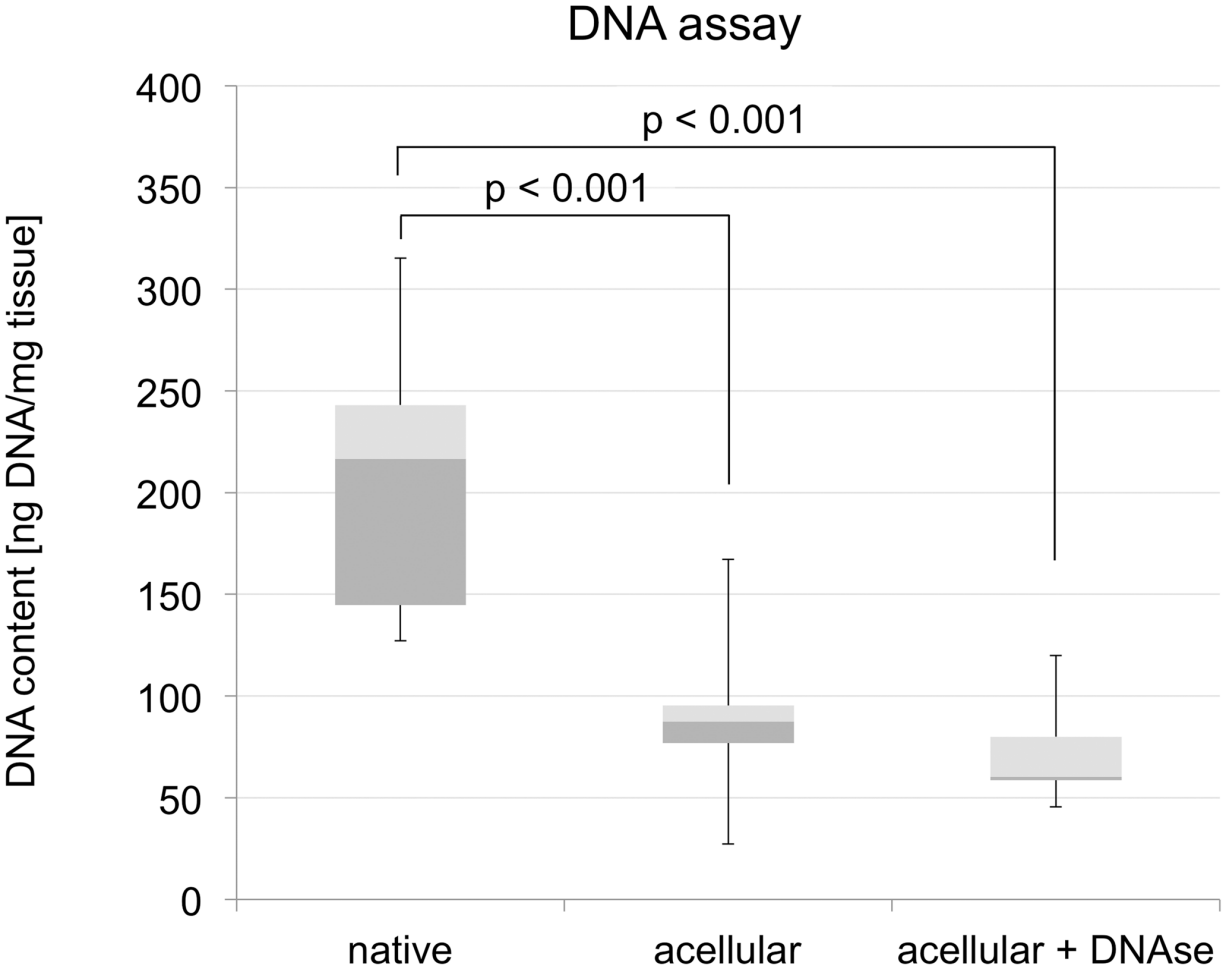
DNA assay of native iliotibial tract samples and corresponding acellular scaffolds. Acellularization related DNA loss was subtotal regardless of the use of DNAse (p<0.001).

**Figure 10:**
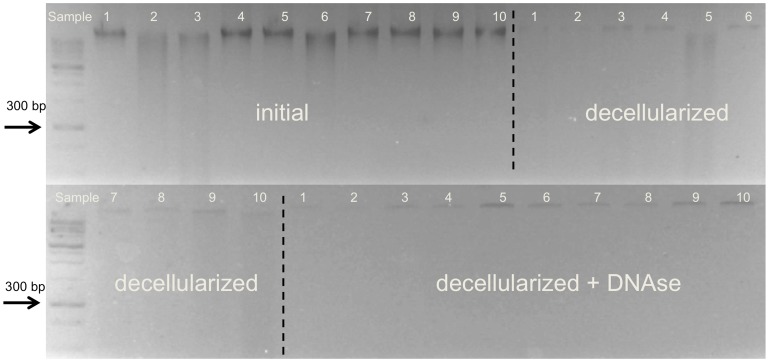
Gel electrophoresis of native iliotibial tract samples and corresponding acellular scaffolds with and without DNAse. Lane numbers indicate the respective sample.

The DNA content of the native tract samples (206±86 ng/mg tissue) was significantly higher than in the acellular scaffolds (92±36 ng/mg tissue; *p*<0.001), indicating a gross but incomplete 55% removal of the DNA (Figure 9). Further treatment of the scaffolds with DNAse caused further non-significant decrease in the DNA content of the acellular tract scaffolds (70±21 ng/mg tissue; 66% removal of the DNA). Qualitative analysis with gel electrophoresis showed intact bands in the native samples with a size larger than 10,000 base pairs (Figure 10). Acellularization caused a gross but incomplete removal of this band, accompanied by a visible DNA-smear below the initial band. When treating the acellular tract scaffolds with DNAse, the smear was removed but the DNA band larger than 10,000 base pairs remained.

## Discussion

This is the first study that directly compares mechanical and histological characteristics of acellular scaffolds and their native counterparts obtained from the same donors and from the same anatomical site. The major aim of this study was to establish a protocol that allows the investigation of minute alterations in the mechanical properties of collagen scaffolds by minimizing potential confounding factors with impact on *ex-vivo* tissue mechanics. The following confounding factors were addressed with potential impact on tensile properties: (1) origin/anatomical site of the tissue, (2) uniaxial testing of tissues that are physiologically loaded uniaxially, (3) same water content and (4) material slippage. On the basis of the protocol, it was furthermore hypothesized that the loss of cells alters the tensile properties in acellular collagen scaffolds such as the iliotibial tract, as compared to the native state. With the given experimental setup, the influence of cells on tissue mechanics is determined indirectly in a model using human iliotibial tract specimens. Tract samples serve as a well-described model of human ligaments and tendons [19], [23], [28], [29].

### 1. Do cells contribute to tensile mechanics of ligaments and tendons?

A comparison of the acellular scaffolds to the native tracts reveals that tensile properties remain largely unchanged, confirming the findings of Hülsmann and coworkers [7] and Pridgen et al. [16]. The elastic modulus and ultimate stress are negligibly affected and the observed deviations of the mean values are partially related to the interindividual variation of the samples. One exception is the maximum strain, which is significantly higher in the acellular tract scaffolds. One explanation for the difference in maximum strain is the following: When the specimen is strained, the collagen bundles are also strained. This strain may cause a lateral (transverse) contraction of the collagen bundles, as shown in our previous study [30]. The described mechanism transfers a load on the partly incompressible fibrocytes or tenocytes, since they are located between the collagen bundles. Thereby the cells contribute to load transfer, as they impose constraints on lateral contraction. If the cells are removed in the acellular scaffolds (Figure 7c,d), the lateral contraction of collagen can slightly continue, explaining the larger extent of tensile strain at maximum stress in the acellular scaffolds. The proposed mechanism might limit the extent of contraction in native samples and therefore collagen elongation. Another explanation might be in terms of a greater interaction between the cells and the matrix. This phenomenon has been reported in the field of material science and research work on reinforced polymers by means of filler materials [31]. Thus, the adhesion between the cells and the matrix in native specimens hinder their elongation and thereby decrease their ductility. Consequently the acellular scaffolds can be strained to further extent until material failure of the extracellular collagen without effects on the elastic modulus or ultimate stress. Beyond these explanations, the washout of extracellular glycosaminoglycans [9], [15] or other negatively charged extracellular matrix proteins could be another explanation [32]–[34] related to the use of SDS or TritonX. However, we found a variable appearance of proteoglycans in both the native and the acellular samples, as indicated by the safranin-o staining (Figure 8). Moreover, Svennson and coworkers found that glycosaminoglycans cannot be considered as mediators of force transmission in human tendons, as their removal does not affect elastic modulus, peak stress and strain [35]. In summary, our static uniaxial measurements indicate that cells do not directly influence tensile tissue mechanics with related stability *in vitro*.

However, fibrocytes or tenocytes play an extensive indirect role in maintaining tissue integrity when loaded mechanically as they are the primary source of the extracellular matrix. The synthesis of collagen and elastin by the cell populations were shown to be dependent upon the load exercised on the cell populations [36]–[38]. Their well-coordinated function is the result of interaction by means of connexins [38], TGFβ [36], [39], [40], matrix metalloproteinases [41], platelet-derived growth factor, tenascin-C [36], bone morphogenetic protein 7 [42], [43] and lysil oxidase [40]. In summary our hypothesis has to be rejected: The role of cells in tendon or ligament biomechanics is largely limited to protein synthesis.

### 2. SDS acellularization negligibly influences uniaxial tensile properties of the human iliotibial tract but incompletely removes DNA from tract scaffolds

To answer the question whether acellular specimens have altered mechanical properties, we established a novel setup taking into account four major issues in the material testing of connective tissues.

First, previous studies used either the native or the acellular condition of tissues obtained from different donors, species or scaffold types [1], [4], [6], [7], [10], [11], [16]. Comparisons based on these data potentially hamper quantifying strain characteristics, as tendons and ligaments are known to vary interindividually. Testing of human tissues was limited to small sample sizes, with the donors' history and other donor related data mostly remaining unclear [1], [7]. Such comparison was not the aim of the aforementioned studies, which focused on a clinical applicability of acellular scaffolds. Secondly, uniaxial stress was previously applied to tissues that are strained multiaxially *in vivo*, e.g. the pericardium [1], [7], [44]. Addressing these issues, we exclusively tested the central part of the human tract in a representative sample size and directly compared tissues from the same donors, combined with histology to verify the extent of acellularization. *In vivo* the central part of the iliotibial tract is mainly exposed to uniaxial strain, which was our rationale for testing it uniaxially to closely resemble the *in-vivo* static situation. The iliotibial tract has no gender- or side-dependent differences [19]. This helped justify our choice of specimens though only a limited number of female specimens was available and mostly from the left side of the human body donors. Presuming that the samples have a rectangular cross section, as done in our study, is another simplification that could potentially influence the results [45]. Third, it is well known that the mechanics of collagen tissue strongly depends on water content [46]–[48]. Altered water content related to the acellularization procedure or interindividual variations, is therefore likely to influence the material testing results to the effect that determining small differences due to acellularization may be hampered. However, the water content was not recorded in previous setups of collagen scaffolds in mechanical testing. To address this issue, we established a protocol using the osmotic stress technique that allows adjusting the water content osmotically in all samples. A fourth issue is material slippage. It frequently occurs in material testing of soft tissues and it introduces unpredictable errors to the results. Sutures or cryoclamping may circumvent this issue but these techniques alter the site of failure with related material properties [11], [44]. Also connective tissues are known to have temperature-dependent strain values [18]. Testing bone-ligament-bone-complexes [6], [11] or partially plastinated samples [19], [23] at a constant temperature is consequently more appropriate. Using the partial plastination method we minimized material slippage and obtained similar tensile data from native samples as published previously [19], [23], [28], [29]. The tensile properties of the acellular scaffolds are also quite similar to the published data [19], [23], [28], [29]. Our findings indicate that SDS does not necessarily impair the tensile properties of collagen tissues at the given concentrations and times, as stated elsewhere [9], [15].

These results and the fact that the acellularization with SDS causes an extensive removal of cellular DNA, shown elsewhere [16], suggest that SDS may have the potential to be used for obtaining acellular scaffolds of ligaments or tendons for surgical reconstruction and reinforcement. However, the microscopic slices revealed that collagen is affected visibly by washout phenomena (Figure 7c,d). Furthermore, the relative extent of DNA removal is less complete in the tract specimens and in tendons [16] when compared to other tissues [8] treated with SDS (Figure 9). Also, DNA fragments of considerable and varying length remain in the scaffolds after acellularization or combined acellularization and further treatment with DNAse, indicated by the smear in gel electrophoresis (Figure 10). The extent of DNA removal complies with a previous study of Pridgen and coworkers using SDS [16]. Recently, the group of Ozasa et al. could show that acellularization with trypsin and Triton X-100 efficiently removes DNA from collagen scaffolds in canine flexor tendons [49]. Triton X-100 might therefore be more suitable for removing the DNA from the collagen scaffolds.

The remaining DNA in acellular scaffolds is suspected to be a target site of immune reactions, e.g. mediated by macrophages [50]. In spite of this theory, commercially available scaffolds contain differing amounts of DNA with differing fragment lengths [51]. As an example, intact DNA was detected in GraftJacket (Wright Medical Technology Inc., Arlington, TN, USA; [51]). Presently there is no uniform standard to evaluate acellular tissues [52] and even cell-free tissues may cause inflammatory reactions, though complete transplant rejection is less likely [51], [53]. Cross-linking of the tissues may help to slow down inflammation [8]. However, this issue was beyond the scope of our study, aiming to determine the mechanical influence of cells on ligament or tendon mechanics. Here, cross-linking would have altered mechanical properties.

The cause for the incomplete removal in the scaffolds, presented here, remains unclear. Possibly, a dense network of collagen species and the undirected matrix of glycosaminoglycans serve as diffusion barriers for both the SDS and the fragmented DNA. The phenomenon of incomplete DNA removal in collagen species was already reported previously [16], [49], [54], [55]. Additional freeze thawing cycles might help enhance the effectiveness of the acellularization [54], but likely damage the tissues mechanically by the formation of ice needles. Furthermore, the group of Adams et al. described a transient decrease of mechanical strength in a canine *in-vivo* model six weeks after surgical implantation of a dermal graft in rotator cuff injuries [10]. It is unclear whether the temporary decrease in mechanical resilience also applies to collagen structures of the human system, though such effects are very likely. One shortcoming of our study is that it does not provide *in-vivo* data of tract samples, which should be addressed in future studies. Further research is necessary to optimize the SDS protocol in collagen scaffolds before clinical application, especially considering the remaining DNA content. Also, the effectiveness of acellularization and the changes of collagen related to SDS should be investigated in more detail, using ultrastructural imaging techniques.

### 3. Clinical implications and outlook

Beyond the academic question whether cells contribute to tensile properties of ligaments and tendons, our study suggests that human iliotibial tract scaffolds may potentially be used for reconstructing ligament tears or tendon ruptures after further improvement of the acellularization protocol with SDS. Mostly, acellular dermal matrices [3]–[5], [12], [56] are used for this purpose, e.g. for abdominal wall [57], [58] or rotator cuff injuries [10], hand reconstruction [11], [12], Achilles tendon rupture [56] or for the hip abductor repair [4]. The rationale behind this application is to reinforce the site of injury [1], [5] – but potentially to the effect that an additional injury is facilitated elsewhere due to the increased stiffness caused by the scaffold. Acellular tissues that closely resemble the healthy condition may circumvent this issue. The tract seems very attractive in this context. It can be obtained easily from (post-mortem) body donors due to its superficial anatomical location and it is an established model of ligament and tendon mechanics. Therefore, acellular tract scaffolds may be an interesting alternative to dermal scaffolds for an anatomical reconstruction in orthopedic or plastic surgery. However, the remaining DNA in the scaffold may cause a scaffold rejection or enhanced inflammatory reactions at the site of surgery. This issue should be solved before tract scaffolds may be applied on patients.

## Supporting Information

Figure S1 **Stress-strain curves of native (left) and acellular (right) iliotibial tract samples.** The relative strain data represent the amount of strain for final cycle until material failure. Same colors are used for the corresponding samples originating from the same body donor. The elastic modulus was determined in the most linear region of the stress-strain curve by means of regression analysis.
(TIF)

Figure S2 **Bland-Altman plots are depicted for the elastic modulus (4a, left), ultimate stress (4b, center) and maximum strain (4c, right).** The mean differences and the upper and lower limits of agreement are depicted. Material properties are unaffected by acellularization with the exception of maximum strain. lLOA  =  lower limit of agreement, mean  =  mean deviation, uLOA  =  upper limit of agreement.
(TIF)

File S1 **This file contains Table S1 and Table S2.** Table S1. Specimen characteristics. Table S2. Mechanical properties, location of failure and statistical data.
(DOCX)
