# Supplementary figures and images for: Do Cells Contribute to Tendon and Ligament Biomechanics?
Source: PLoS One. 2014 Aug 15;9(8):e105037. doi: 10.1371/journal.pone.0105037 (PMC4134275; doi:10.1371/journal.pone.0105037)

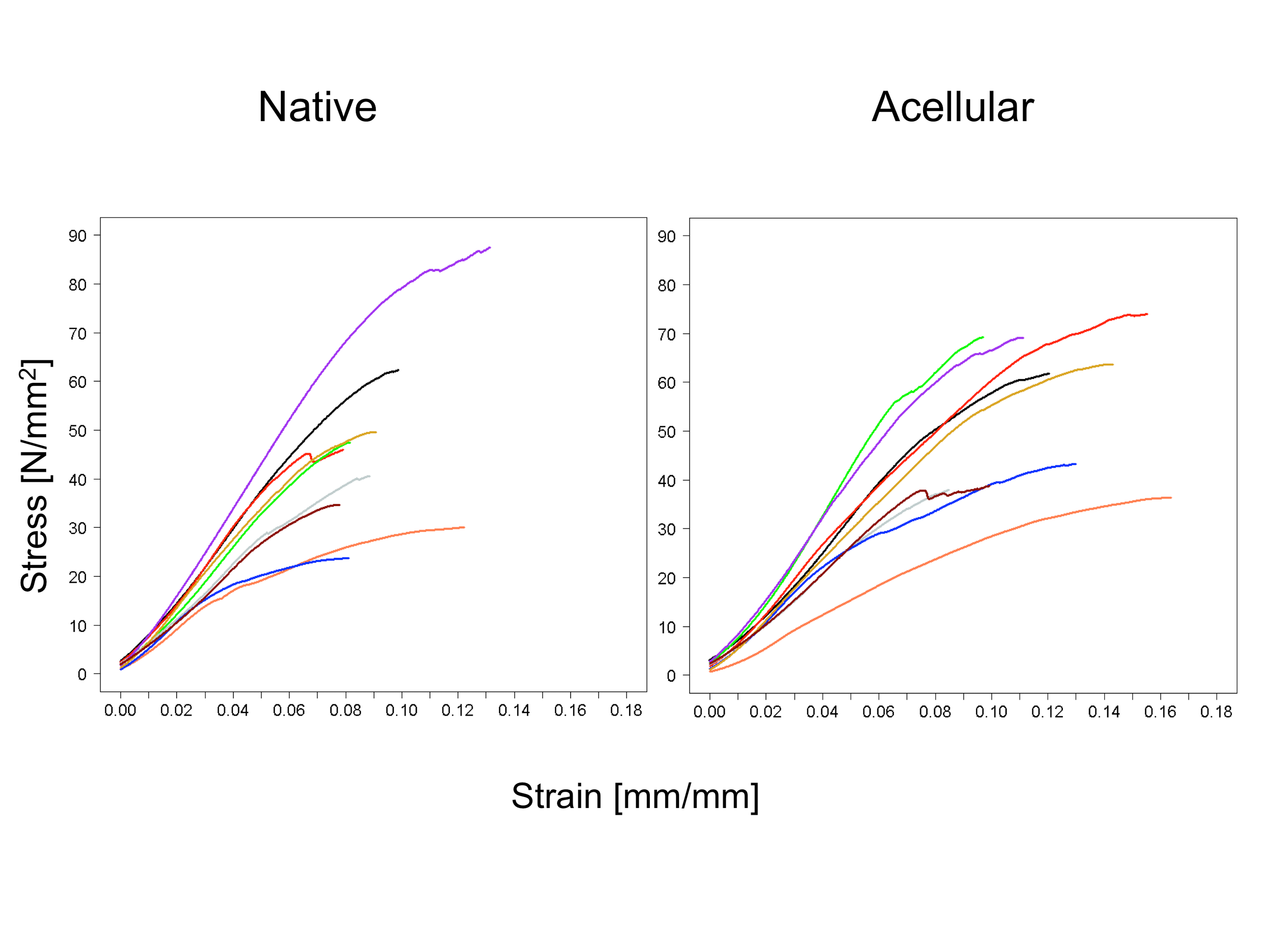

Supplement: Figure S1 — Stress-strain curves of native (left) and acellular (right) iliotibial tract samples. The relative strain data represent the amount of strain for final cycle until material failure. Same colors are used for the corresponding samples originating from the same body donor. The elastic modulus was determined in the most linear region of the stress-strain curve by means of regression analysis. (TIF) [file pone.0105037.s001.tif]

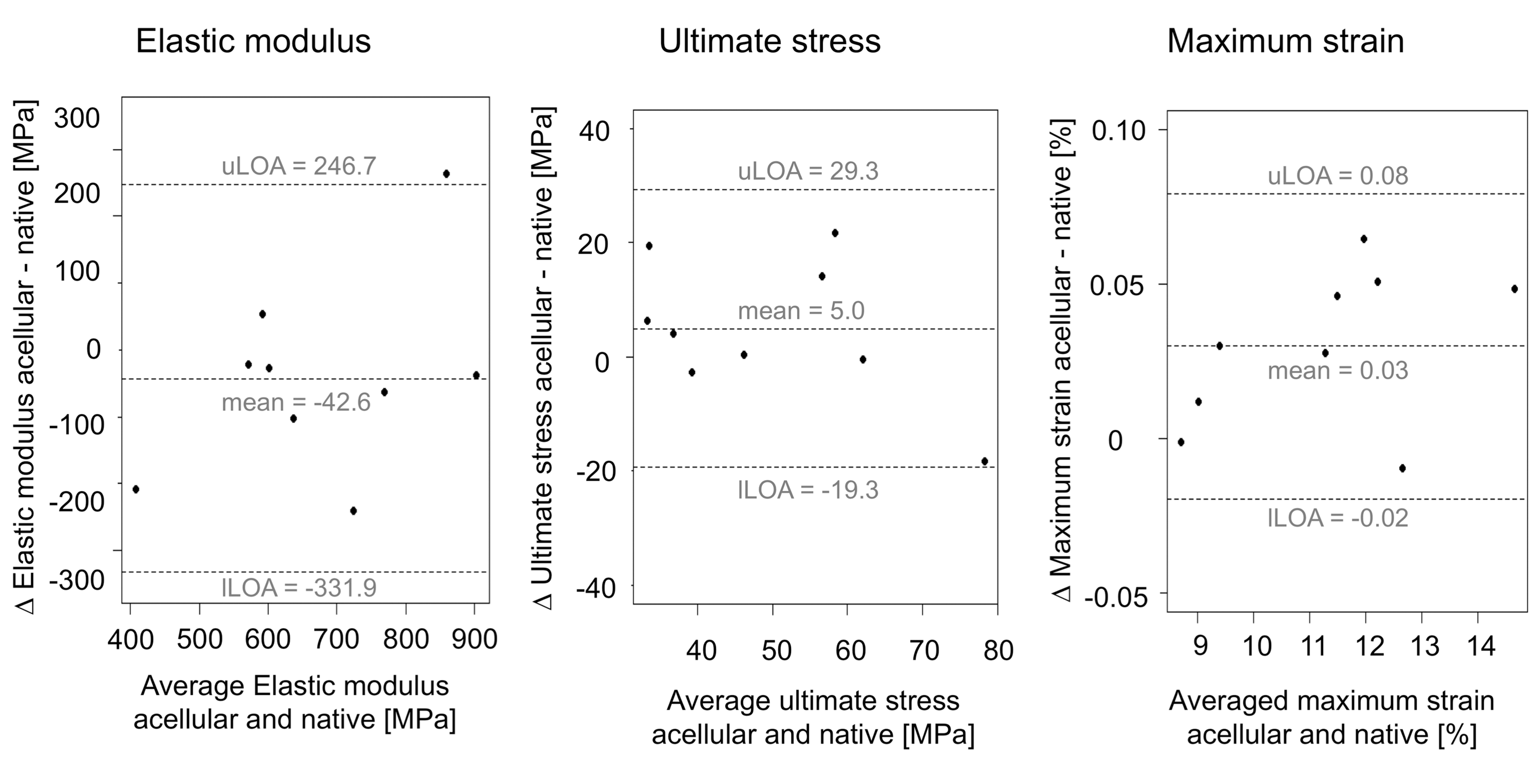

Supplement: Figure S2 — Bland-Altman plots are depicted for the elastic modulus (4a, left), ultimate stress (4b, center) and maximum strain (4c, right). The mean differences and the upper and lower limits of agreement are depicted. Material properties are unaffected by acellularization with the exception of maximum strain. lLOA = lower limit of agreement, mean = mean deviation, uLOA = upper limit of agreement. (TIF) [file pone.0105037.s002.tif]
