# Supplementary material for: Do Cells Contribute to Tendon and Ligament Biomechanics?
Source: PLoS One. 2014 Aug 15;9(8):e105037. doi: 10.1371/journal.pone.0105037 (PMC4134275; doi:10.1371/journal.pone.0105037)
Supplement: File S1 — This file contains Table S1 and Table S2. Table S1. Specimen characteristics. Table S2. Mechanical properties, location of failure and statistical data. (DOCX) [file pone.0105037.s003.docx]

*Table S1: Specimen characteristics of iliotibial tract specimens*

| **Specimen** | **Gender** | **Age** | **Body length** | **Body weight** | **Specimen** | **Cause of death** |
| --- | --- | --- | --- | --- | --- | --- |
| **number** |  | **[years]** | **[cm]** | **[kg]** | **side** |  |
|  |  |  |  |  |  |  |
| 1 | male | 28 | 196 | 104 | left | Suicide by hanging |
| 2 | male | 36 | 182 | 93 | left | Headshot |
| 3 | male | 33 | 174 | 82 | left | Hemorrhage |
| 4 | male | 29 | 170 | 51 | left | Diabetic coma |
| 5 | male | 18 | 185 | 108 | left | Suicide by hanging |
|  |  |  |  |  |  |  |
| **Mean** |  | **28.80** | **181.40** | **87.60** |  |  |
| **Standard deviation** | | **6.11** | **9.07** | **20.42** |  |  |
|  |  |  |  |  |  |  |
| **Specimen** | **Gender** | **Age** | **Body length** | **Body weight** | **Specimen** | **Cause of death** |
| **number** |  | **[years]** | **[cm]** | **[kg]** | **side** |  |
|  |  |  |  |  |  |  |
| 6 | male | 24 | 162 | 53 | left | Suicide by hanging |
| 7 | female | 25 | 168 | 57 | right | Hemothorax |
| 8 | male | 20 | 188 | 117 | left | Polytrauma |
| 9 | male | 34 | 189 | 68 | left | Hemorrhage |
| 10 | male | 76 | 185 | 106 | right | Hemorrhage |
| 11 | male | 25 | 182 | 69 | left | Undercooling |
| 12 | female | 54 | 161 | 61 | right | Brain death |
| 13 | female | 29 | 171 | 58 | left | Hemorrhage |
| 14 | female | 83 | 151 | 55 | left | Hemorrhage |
|  |  |  |  |  |  |  |
| **Mean** |  | **41.11** | **173.00** | **71.56** |  |  |
| **Standard deviation** | | **22.59** | **12.84** | **22.10** |  |  |
|  |  |  |  |  |  |  |
| **Specimen** | **Gender** | **Age** | **Body length** | **Body weight** | **Specimen** | **Cause of death** |
| **number** |  | **[years]** | **[cm]** | **[kg]** | **side** |  |
|  |  |  |  |  |  |  |
| 15 | male | 50 | 188 | 85 | left | Traumatic brain injury |
| 16 | male | 20 | 186 | 93 | left | Polytrauma |
| 17 | male | 25 | 184 | 90 | left | Polytrauma |
| 18 | male | 29 | 185 | 65 | left | Hemorrhage |
| 19 | female | 33 | 175 | 65 | left | Drug intoxication |
| 20 | male | 31 | 184 | 90 | left | Traumatic brain injury |
| 21 | female | 22 | 167 | 50 | left | Diabetic coma |
| 22 | male | 20 | 178 | 107 | left | Polytrauma |
| 23 | male | 32 | 182 | 96 | left | Hypoxic brain damage |
| 24 | male | 32 | 174 | 78 | left | Subarachnoid hemorrhage |
|  |  |  |  |  |  |  |
| **Mean** |  | **29.11** | **181.00** | **82.33** |  |  |
| **Standard deviation** | | **8.39** | **6.28** | **16.42** |  |  |

*Table S2: Mechanical properties, failure location and related statistical data of iliotibial tract specimens*

|  |  |  |  |  |  |  |  |  |
| --- | --- | --- | --- | --- | --- | --- | --- | --- |
| **Specimen** | **Young's modulus [MPa]** | | **Ultimate stress [MPa]** | | **Maximum strain [%]** | | **Location of failure** | |
| **number** | native | acellular | native | acellular | native | acellular | native | acellular |
|  |  |  |  |  |  |  |  |  |
|  |  |  |  |  |  |  |  |  |
| 6 | 800.5 | 737.6 | 62.3 | 61.8 | 9.9 | 12.7 | central | central |
| 7 | 844.2 | 604.4 | 45.9 | 46.4 | 8.7 | 15.2 | central | central |
| 8 | 512.2 | 303.1 | 30.1 | 36.4 | 12.2 | 17.1 | central | central |
| 9 | 614.6 | 588.3 | 40.6 | 38.0 | 8.8 | 8.6 | central | central |
| 10 | 565.0 | 618.8 | 23.7 | 43.3 | 9.2 | 13.8 | central | cranial |
| 11 | 921.2 | 882.7 | 87.4 | 69.1 | 13.1 | 12.2 | central | central |
| 12 | 687.6 | 585.2 | 49.7 | 63.7 | 9.7 | 14.8 | central | central |
| 13 | 581.7 | 560.6 | 34.7 | 38.8 | 7.9 | 10.9 | cranial | central |
| 14 | 726.7 | 990.1 | 47.4 | 69.2 | 8.4 | 9.6 | central | central |
|  |  |  |  |  |  |  |  |  |
|  |  |  |  |  |  |  |  |  |
| **Recorded mean value** | **694.9** | **652.3** | **46.9** | **51.9** | **9.8** | **12.9** |  |  |
| **Standard deviation** | **139.3** | **199.0** | **19.0** | **13.9** | **1.7** | **2.6** |  |  |
|  |  |  |  |  |  |  |  |  |
| **P value** | **0.412** | | **0.262** | | **0.005** | |  |  |
|  |  |  |  |  |  |  |  |  |
